# Preclinical study on an adsorbed acellular diphtheria- tetanus-pertussis (reduced-dose) combined vaccine: evaluation of reproductive safety and immunogenicity

**DOI:** 10.3389/fimmu.2026.1828345

**Published:** 2026-05-26

**Authors:** Han Chu, Wenlu Kong, Qin Gu, Yan Ma, Lukui Cai, Jingyan Li, Xiaoyu Wang, Qiuyan Ji, Jiana Wen, Na Gao, Guang Ji, Wenzhu Hu, Ting Zhao, Ling Ping, Yuting Fu, Ying Li, Tiannan Gong, Yingfu Jin, Yufan Yang, Yixian Fu, Xinhua Qin, Huimei Zheng, Jingsi Yang, Jiangli Liang

**Affiliations:** 1 Institute of Medical Biology, Chinese Academy of Medical Sciences & Peking Union Medical College, Kunming, China; 2 State Key Laboratory of Respiratory Health and Multimorbidity, Beijing, China; 3 Yunnan Provincial Biopharmaceutical Translation and Evaluation Technology Innovation Center, Kunming, China; 4 Kunming Scientific and Technological Innovation Center for R&D and Industrialization of Vaccines Against Novel and Emerging Highly Pathogenic Pathogens, Kunming, China

**Keywords:** diphtheria, tetanus and acellular pertussis combined vaccine (DTaP), immunogenicity, repeated dose toxicity, reproductive safety, safety evaluation

## Abstract

**Introduction:**

The diphtheria, tetanus, and acellular pertussis combined vaccine (DTaP) is a trivalent formulation containing acellular pertussis antigens, diphtheria toxoid, and tetanus toxoid, and provides simultaneous protection against pertussis, diphtheria, and tetanus. This study aimed to systematically evaluate the effects of repeated intramuscular administration of an adsorbed acellular diphtheria-tetanus-pertussis (reduced-dose) combined vaccine (Tdacp) on gamete maturation, mating performance, fertility, and embryo-fetal development in Sprague-Dawley (SD) rats, thereby providing experimental evidence for its reproductive safety.

**Methods:**

Sexually mature SD rats were randomly assigned, stratified by sex and body weight into four groups: negative control, adjuvant control, low-dose, and high-dose, with 40 rats per group (20 males and 20 females). Male and female rats were paired for mating according to group assignment and identification number. Animals received two intramuscular injections; the low-dose group received 0.25 mL/rat, whereas all other groups received 0.5 mL/rat. Evaluated parameters included clinical signs, food consumption, body weight, reproductive organ coefficients, estrous cycle characteristics in females, sperm parameters in males, embryo-fetal growth and development, and vaccine-induced antibody responses.

**Results:**

Repeated intramuscular administration of Tdacp did not result in significant adverse effects on food consumption, body weight, or reproductive function in either the low-dose or high-dose group compared with the negative control group. Likewise, no treatment-related embryo-fetal developmental toxicity or teratogenic effects were observed. Although several embryo-fetal developmental parameters in the adjuvant control group, including implantation loss, fetal viability, embryo/fetal resorption, and selected skeletal ossification findings, differed from those in the negative control group, similar findings were not observed in the vaccine-treated groups. Immunogenicity analysis demonstrated positive antibody responses against diphtheria, tetanus, and pertussis antigens in both vaccine groups following immunization.

**Discussion:**

These findings indicate that Tdacp has a favorable reproductive safety profile and good immunogenicity in SD rats. The results provide important nonclinical evidence to support its further clinical development. Manufactured using advanced chromatographic purification technology, this combined vaccine may contribute to the establishment and implementation of a life-course pertussis prevention and immunization strategy in China.

## 1 Introduction

Pertussis, diphtheria, and tetanus are significant vaccine-preventable diseases worldwide. Among them, pertussis is a highly contagious respiratory disease caused by Bordetella pertussis (1). Despite high vaccination coverage in many countries, pertussis continues to occur periodically, and a resurgence of cases has been observed in recent years (2). Notably, the incidence of pertussis among adolescents and adults has increased, and these populations have become important sources of transmission to infants (3). This epidemiological shift has increased the risk of severe disease and mortality in newborns, highlighting the ongoing challenges in pertussis prevention and control.

In China, pertussis prevention within the national immunization program relies on DTaP vaccines rather than standalone pertussis vaccines. These vaccines are predominantly produced using co-purification processes. Although the co-purification process is well established, it presents certain limitations with respect to antigen composition control, antigen content adjustment, and batch-to-batch consistency. In contrast, component acellular pertussis vaccines allow for more precise control of antigen composition and dosage and have been widely adopted in many countries. Currently, pertussis-containing vaccines licensed in China are primarily intended for pediatric use, and vaccines specifically designed for adolescents, adults, and pregnant women remain limited (4). As a result, infants may lack early protection and remain vulnerable to infection, particularly from household contacts with waning immunity (5). In this context, maternal immunization during pregnancy has been recognized as an effective strategy to provide passive protection to infants through transplacental antibody transfer (6–8).

The Tdacp vaccine evaluated in the present study is an component acellular pertussis-containing vaccine manufactured using column chromatography-based purification to ensure well-defined antigen composition, high purity, and consistent antigen content. This vaccine is intended for booster immunization in adolescents and adults, with particular relevance for women of childbearing age and pregnant women, to provide direct protection to the mother and passive protection to the newborn through maternal immunization. The vaccine is formulated with an aluminum-based adjuvant, and preclinical studies have demonstrated favorable immunogenicity and an acceptable safety profile (9, 10), supporting its potential use in these populations. Similar vaccines have been incorporated into booster and maternal immunization programs in several countries (11). However, no comparable vaccine has yet been approved in China.

According to the International Council for Harmonisation (ICH) guideline S5(R3) on the detection of toxicity to reproduction for human pharmaceuticals, vaccines intended for use in pregnant women, women of childbearing age, or populations with repeated exposure are recommended to undergo systematic reproductive toxicity evaluation. Therefore, the present study systematically evaluated the reproductive toxicity and immunogenicity of the Tdacp vaccine following repeated intramuscular administration in male and female rats, as well as in pregnant rats, using a standardized animal model. The results provide important nonclinical safety data to support subsequent clinical studies and offer critical toxicological evidence for the development and regulatory evaluation of pertussis-containing vaccines suitable for adolescents, adults, and pregnant women in China.

## 2 Materials and methods

### 2.1 Test animals and groups

Specific-pathogen-free (SPF) SD rats were obtained from Beijing Vital River Laboratory Animal Technology Co., Ltd. (Production License No. SCXK (Jing) 2021-0006, issued by the Beijing Municipal Science and Technology Commission). The animal quality certificate numbers were No. 110011241104543013 and No. 110011241104543175. A total of 160 rats (80 males and 80 females) were used in this study. At study initiation, male rats weighed 290.1-336.7g and female rats weighed 186.9-231.1g.

All animals were housed in a barrier facility under controlled environmental conditions, with a temperature of 20-26°C and relative humidity of 40%-70%, and were provided with food and water ad libitum. Before study initiation, all animals underwent a 3-day quarantine period followed by a 7-day acclimation period. Quarantine observations included general appearance, body conformation, activity, respiration, coat condition, nose, oral cavity, eyes, ears, genitalia, urine, and feces. The study protocol was reviewed and approved by the Institutional Animal Care and Use Committee (IACUC) of Shandong Xinbo Pharmaceutical Research Co., Ltd. (Approval No. XB-IACUC-20240983).

Using TOXSTAT2006 software, the 160 sexually mature SD rats were randomly assigned, stratified by sex and body weight, into four groups: negative control, adjuvant control, low-dose, and high-dose groups, with 20 males and 20 females in each group. For mating, females were paired with males within the same treatment group according to animal identification number. Prior to mating, vaginal smears were collected from female rats to determine the stage of the estrous cycle. Females identified as being in proestrus were then co-housed with males at a ratio of 1:1 overnight. The following morning, vaginal plugs were checked, and for females without a visible plug, vaginal smears were examined for the presence of sperm. Detection of either a vaginal plug or sperm was considered evidence of successful mating. Females with confirmed mating were considered pregnant, and the day of mating confirmation was designated as gestation day 0 (GD 0).

### 2.2 Vaccine

The vaccine used in this study was an adsorbed component acellular diphtheria, tetanus, and pertussis (reduced-dose) combined vaccine (Batch No.: 20240301), independently developed and produced by the Institute of Medical Biology, Chinese Academy of Medical Sciences, with a dosage of 0.5 ml per dose. Each dose contains diphtheria toxoid (2 Lf), tetanus toxoid (2.5 Lf), pertussis toxin (8 μg), filamentous hemagglutinin (8 μg), and pertactin (2.5 μg), with 1.2 mg of aluminum hydroxide used as an adjuvant. Additionally, the adjuvant control was aluminum hydroxide, and the negative control was 0.9% sodium chloride injection, both prepared by the Institute of Medical Biology, Chinese Academy of Medical Sciences.

All administrations were performed by intramuscular injection into the medial aspect of the hind limbs. Dose levels were selected on the basis of the intended single clinical human dose of the investigational Tdacp vaccine, which is 0.5 mL per dose. Accordingly, the high-dose group received 1 human dose per animal, corresponding to 0.5 mL/animal, whereas the low-dose group received 0.5 human dose per animal, corresponding to 0.25 mL/animal. The adjuvant control and negative control groups both received 0.5 mL/animal to maintain consistency of administration volume with the high-dose group and to support interpretation of reproductive and developmental toxicity findings within the overall study design. To ensure comparable injection conditions, the negative control and adjuvant control groups received 0.5 mL/animal, matching the volume used in the high-dose group. Male rats were dosed twice, at 3 weeks and 1 week before mating, and female rats were dosed twice, at 1 week before mating and on GD 6. In the high-dose, negative control, and adjuvant control groups, 0.5 mL/animal was administered bilaterally into both hind limbs (0.25 mL/site). In the low-dose group, 0.25 mL/animal was administered as a single 0.25 mL injection, with the injection site alternated between the left and right hind limbs. Animals in the high-dose and low-dose groups received Tdacp, whereas animals in the negative control group and adjuvant control group received the corresponding control formulations.

### 2.3 Observations and measurements

During the study period, all animals were observed daily for general clinical condition. On each dosing day, an additional observation was performed within 1 h after administration. Local reactions at the injection site, including erythema, edema, hyperemia, and swelling, were assessed daily after dosing.

Before mating, body weight was measured twice weekly and 24-h food consumption was recorded once weekly for both male and female rats; these parameters were not assessed during the mating period. After mating, pregnant females were weighed and their food consumption was recorded on GDs 0, 3, 6, 9, 12, 15, 18, and 20. Male rats were weighed once before necropsy.

After mating, blood samples were collected from eight male rats per group, except those in the adjuvant control group, for antibody analysis. The adjuvant control group was not included in the antibody analysis because the adjuvant-only formulation did not contain diphtheria, tetanus, or pertussis antigens and therefore was not expected to induce the corresponding antigen-specific IgG responses; the negative control group served as the background baseline for the assay. Serum IgG antibodies against pertussis, diphtheria, and tetanus were measured by enzyme-linked immunosorbent assay (ELISA). At necropsy, the testes, epididymides, and left cauda epididymidis were weighed. Epididymal sperm count, sperm morphology (by smear examination), and sperm motility were then evaluated. In addition, all animals underwent a gross examination of external features and major organs/tissues. The testes, epididymides, prostate, and seminal vesicles were collected and preserved for further analysis.

**Figure 1:**
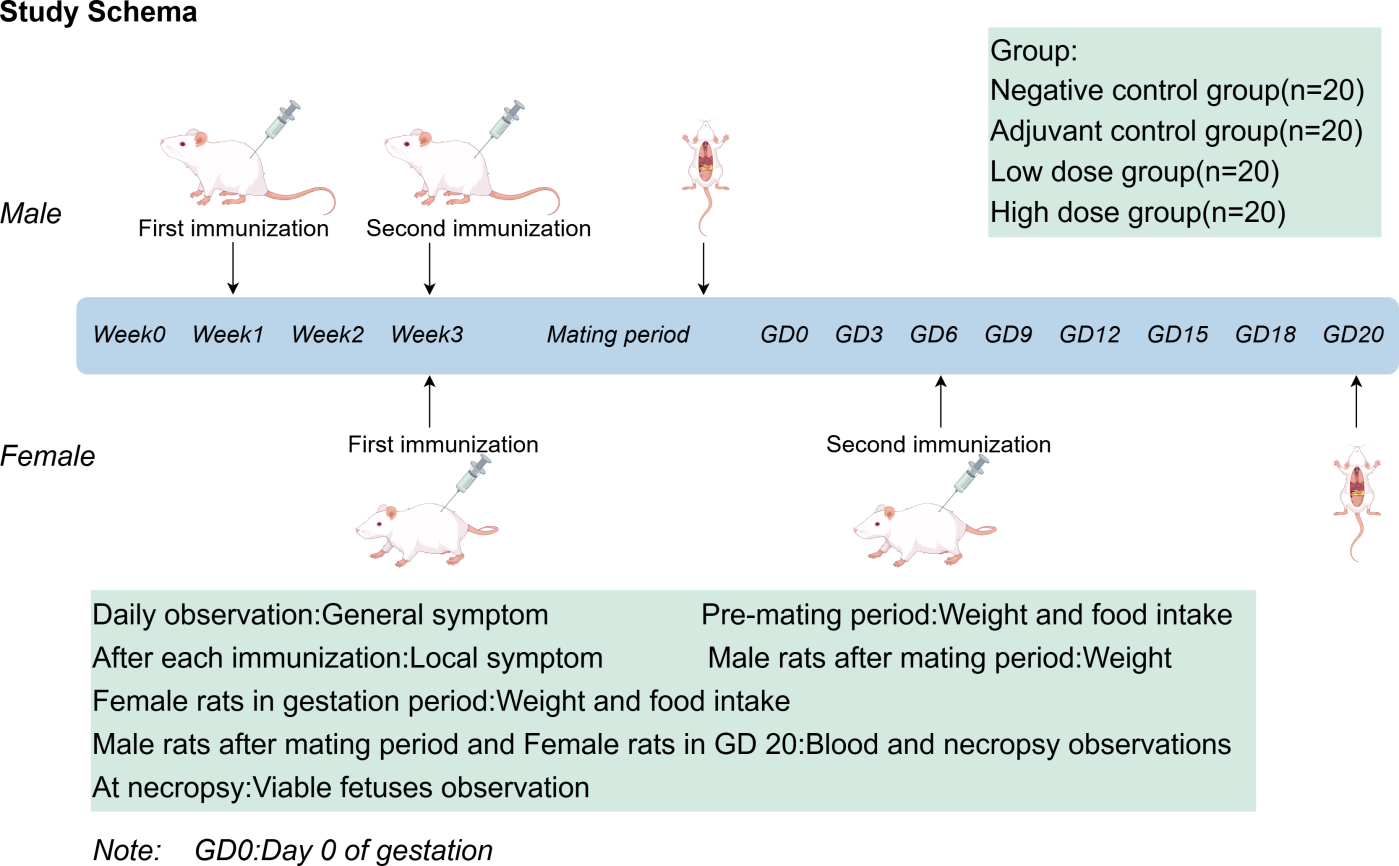
Study schema. All the rats completed two immunizations within the specified intervals and the relevant indicators were tested at the predetermined time points.

On GD 20, blood samples were collected from eight pregnant females per group, except those in the adjuvant control group, together with samples from their corresponding litters, for determination of antibody levels. The females were then necropsied to confirm pregnancy status. A gross examination of major organs and tissues was performed, and the uterus, vagina, and ovaries were collected. The numbers and status of implantation sites were recorded, and the uterus was opened to assess live fetuses. Fetuses were classified accordingly, and fetal visceral examinations by sectioning and skeletal examinations were performed in the high-dose, negative control, and adjuvant control groups (**Figure 1**).

### 2.4 Statistical analysis

Categorical data, including mortality, premature delivery, abortion, incidence of clinical signs, male mating and fertility indices, and female conception and pregnancy indices, were analyzed using Fisher’s exact test. Sex ratio was compared using Pearson’s chi-square test. Continuous data, including pre-mating body weight and food consumption, were first assessed for homogeneity of variance using Bartlett’s test. Data with homogeneous variance (*P*≥0.05) were analyzed by one-way analysis of variance (ANOVA), followed by Dunnett’s test when the overall comparison was statistically significant. Data with heterogeneous variance (*P* < 0.05) were analyzed using the Kruskal-Wallis test, followed by appropriate nonparametric multiple-comparison procedures where applicable. Fetal and developmental abnormality data, including dead fetus rate and the rates of external, skeletal, and visceral malformations, were analyzed using both litter-based rate analysis and incidence-based analysis. In the litter-based analysis, percentages were compared using the Kruskal-Wallis H test, followed by the Nemenyi test for multiple comparisons when appropriate, or the Wilcoxon rank-sum test for pairwise comparisons. In the incidence-based analysis, Fisher’s exact test was used to compare the frequencies of abnormal litters, abnormal fetuses, and live fetuses between groups. All *post hoc* comparisons were one-tailed. A *P* value<0.05 was considered statistically significant, and *P* < 0.01 was considered highly significant.

## 3 Results

### 3.1 Post-dose clinical observations and changes in body weight and food consumption

**Figure 2:**
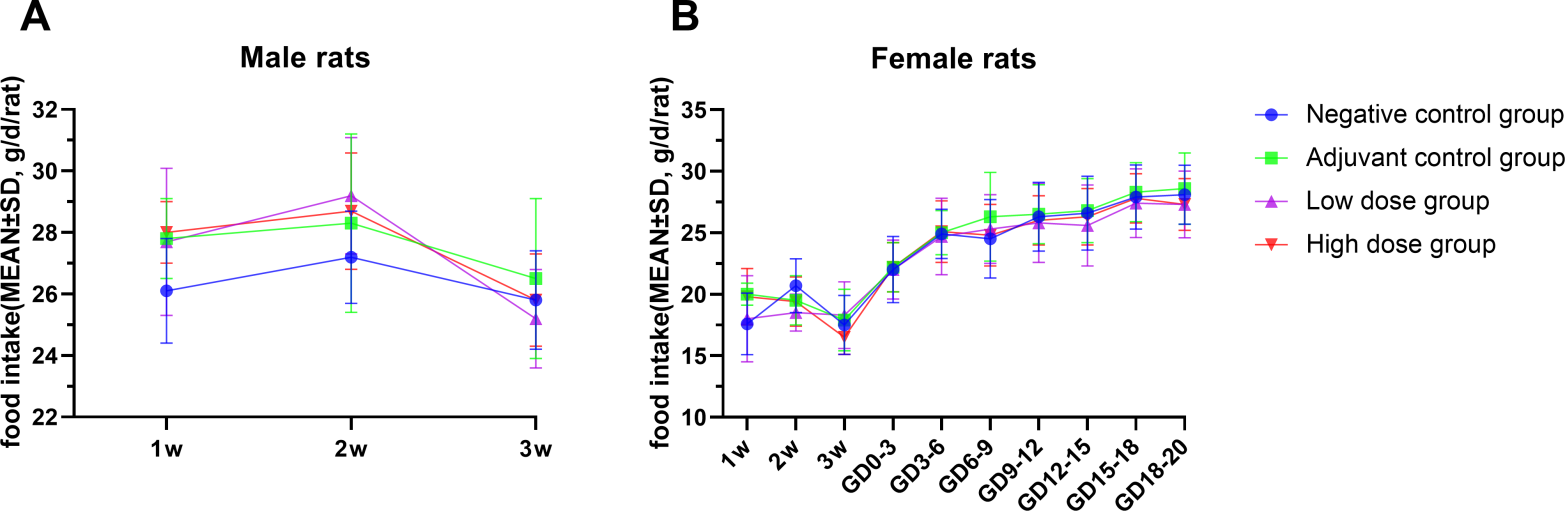
Changes in food intake during premating, mating, and gestation periods in SD rats treated with Tdacp. **(A)** male rats. **(B)** female rats.

**Figure 3:**
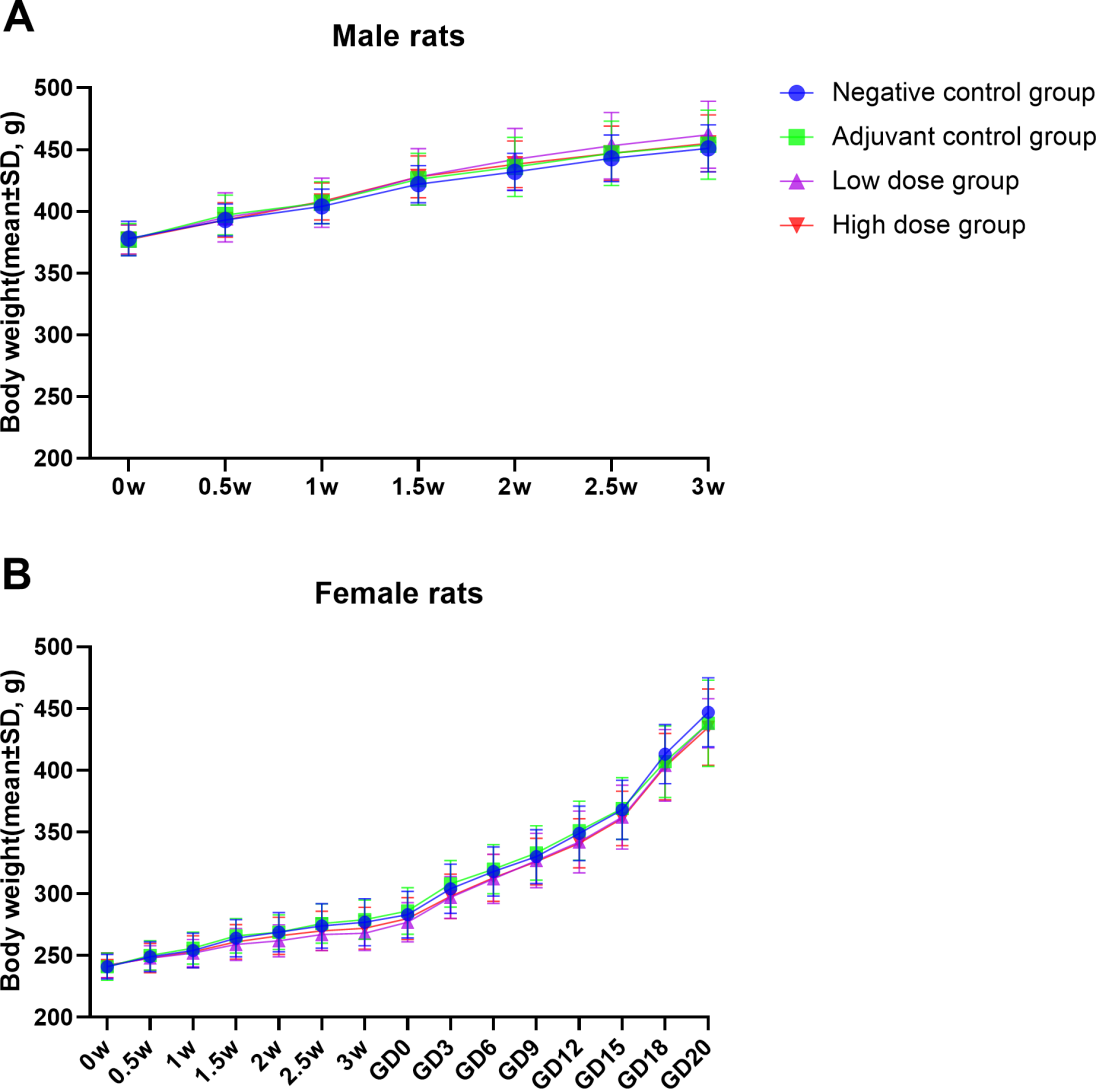
Changes in body weight during premating, mating, and gestation periods in SD rats treated with Tdacp. **(A)** male rats. **(B)** female rats.

Mating was successful in all groups, with a mating rate of 100% (20/20). No mortality, moribundity, abortion, or premature delivery was observed in any group. No treatment-related abnormalities were noted during post-dose clinical observations. Food consumption (**Supplementary Table 1**; **Figure 2**) and body weight (**Supplementary Table 2**; **Figure 3**) in the high-dose group, low-dose group, and adjuvant control group did not differ significantly from those in the negative control group (*P*≥0.05).

### 3.2 Male fertility

#### 3.2.1 Reproductive organ weights and organ-to-body weight ratios

**Figure 4:**
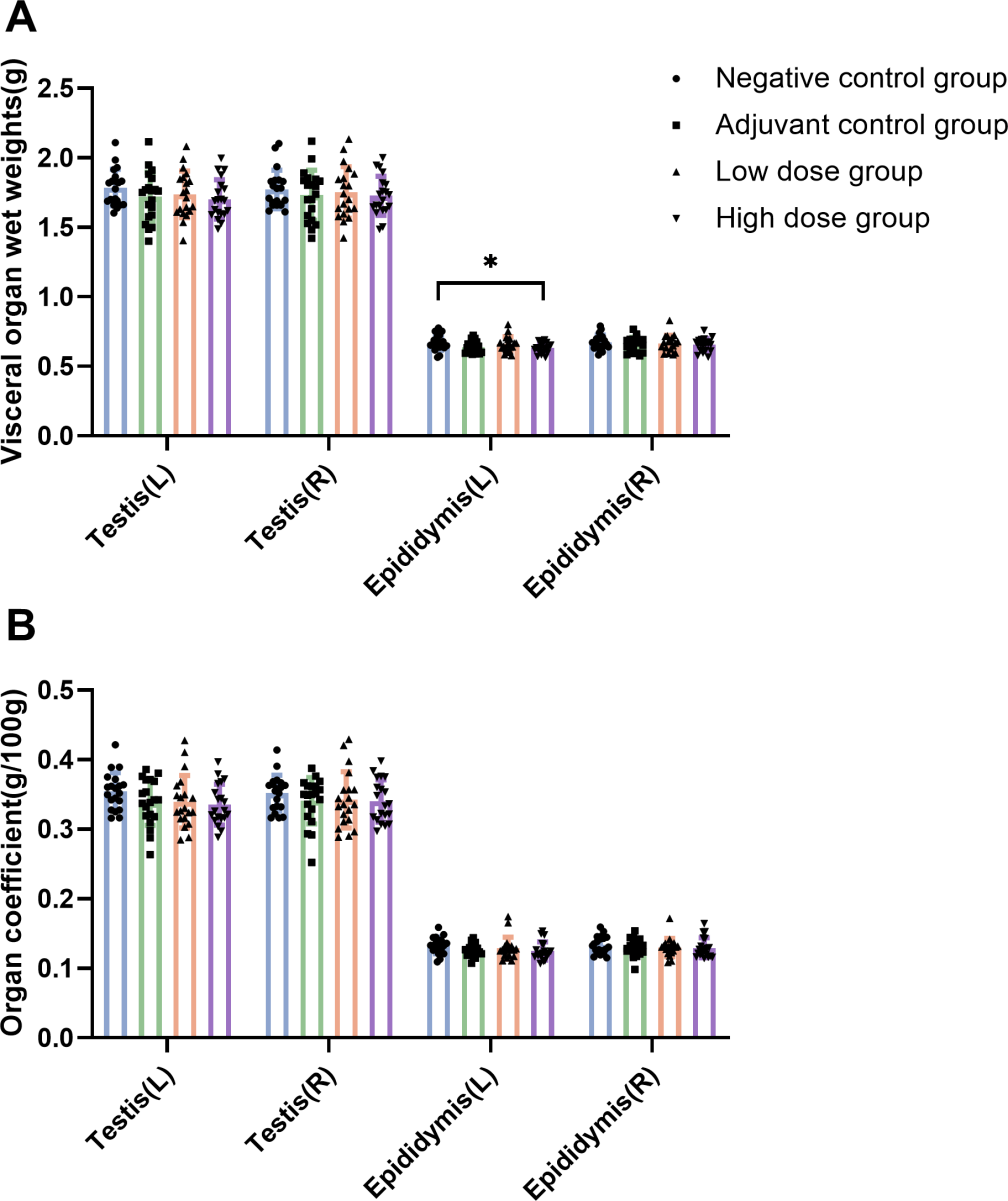
Reproductive organ wet weights and organ-to-body weight ratios in male rats. **(A)** visceral organ wet weights. **(B)** organ coefficient. A t-test was used for comparison of visceral organ wet weights. Statistical significance was indicated as follows: *P<0.05.

The wet weight of the left epididymis was significantly lower in the high-dose group than in the negative control group (*P* < 0.05). This finding was considered incidental and unrelated to the test article, as it was likely due to the relatively high value in the negative control group. No other statistically significant abnormalities were observed in reproductive organ weights or organ-to-body weight ratios (**Supplementary Table 3**; **Figure 4**).

#### 3.2.2 Epididymal sperm count, motility, and morphology

No statistically significant differences were observed in epididymal sperm count, sperm motility, or sperm abnormality rate in the high-dose, low-dose, or adjuvant control group compared with the negative control group (*P*≥0.05) (**Supplementary Table 4**).

### 3.3 Female fertility

#### 3.3.1 Estrous cycle examination

No statistically significant differences in estrous cycle parameters were observed in the high-dose, low-dose, or adjuvant control group compared with the negative control group (*P*≥0.05) (**Supplementary Table 5**).

#### 3.3.2 Litter weight and ovarian parameters in pregnant rats

**Figure 5:**
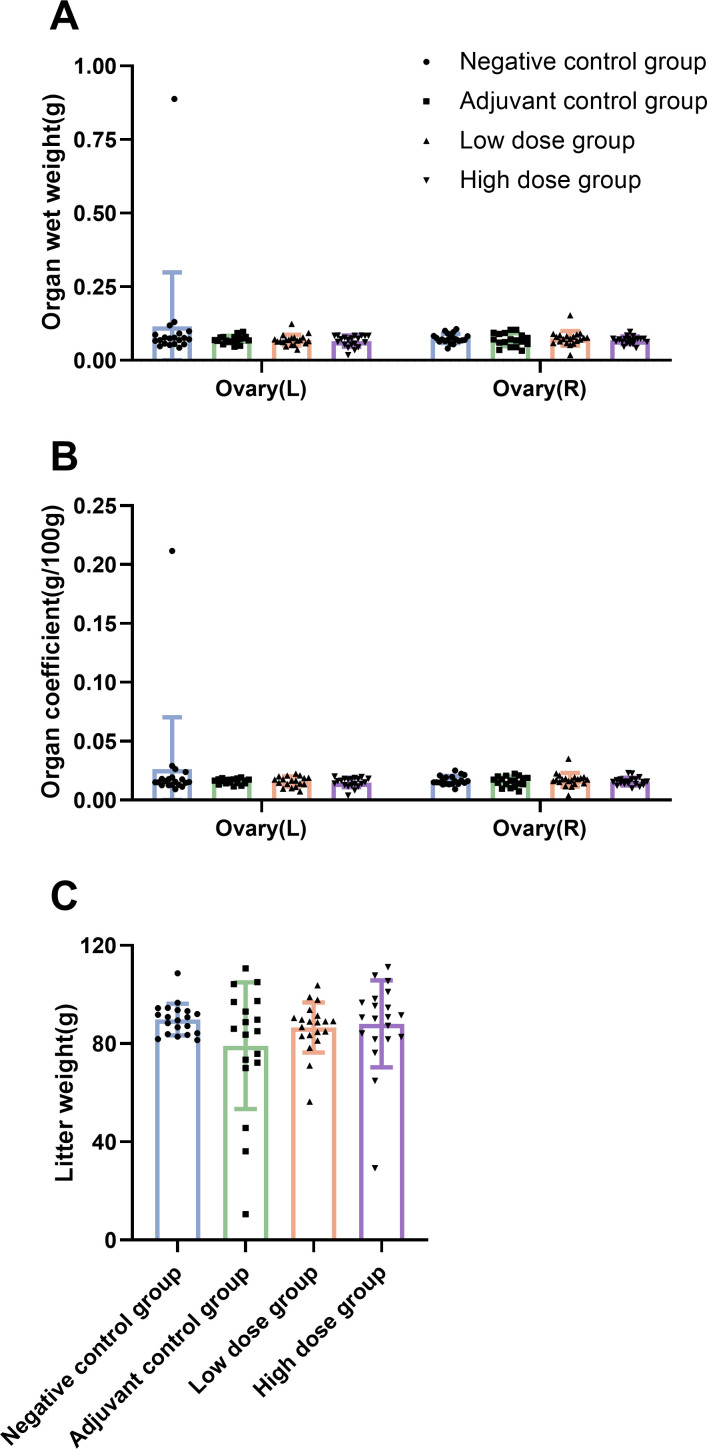
Ovarian wet weight, organ-to-body weight ratio, and litter weight in Pregnant Rats. **(A)** organ wet weights. **(B)** organ coefficient. **(C)** litter weight.

No statistically significant differences were observed in litter weight, left and right ovarian weights, or ovarian-to-body weight ratios in the high-dose, low-dose, or adjuvant control group compared with the negative control group (*P*≥0.05). Although one animal in the negative control group showed a relatively high ovarian weight and ovarian-to-body weight ratio, this finding was considered an isolated individual variation and was not associated with any other abnormal findings. (**Supplementary Table 3**; **Figure 5**).

#### 3.3.3 Corpora lutea, implantation sites, live fetuses, dead fetuses, and pre- and post-implantation loss in pregnant rats

The adjuvant control group showed significantly higher mean numbers of dead fetuses, pre-implantation loss, and post-implantation loss than the negative control group (*P* < 0.05). However, the corresponding values in the high-dose group were lower than those in the adjuvant control group. Moreover, no statistically significant differences were observed between the adjuvant control group and negative control group in the numbers of implantation sites or live fetuses (*P*≥0.05). These findings were not accompanied by other abnormal changes in reproductive or embryo-fetal developmental parameters. Therefore, although changes were observed in the adjuvant control group, they were not considered to indicate a clear treatment-related toxicological effect under the conditions of this study (**Table 1**).

**Table 1:** Number of corpora lutea, implantation sites, live and dead fetuses, and pre- and post-implantation loss in pregnant rats.

Parameters	Negative control group (n=20)	Adjuvant control group (n=18)			Low dose group (n=20)			High dose group (n=20)		
			χ^2^	*P*		χ^2^	*P*		χ^2^	*P*
Number of Corpora Lutea	324	299	0.481	0.933	325	0.060	>0.999	324	<0.001	>0.999
Mean Number of Corpora Lutea(MEAN ± SD)	16.2 ± 1.4	16.6 ± 3.8			16.3 ± 2.7			16.2 ± 2.2		
Number of Implantation Sites	314	263	1.179	0.506	306	0.445	0.946	301	0.723	0.813
Mean Number of Implantations(MEAN ± SD)	15.7 ± 1.2	14.6 ± 4.3			15.3 ± 1.9			15.1 ± 3.1		
Number of Live Fetuses	311	249	1.764	0.197	298	0.686	0.835	299	0.633	0.864
Mean Number of Live Fetuses(MEAN ± SD)	15.6 ± 1.1	13.8 ± 4.7			14.9 ± 2.0			15.0 ± 3.2		
Dead Fetuses(MEAN ± SD)	0.2 ± 0.4	0.8 ± 1.0**	3.244	0.005	0.4 ± 0.5	1.327	0.412	0.1 ± 0.3	0.265	0.987
Pre-implantation Loss (%)	2.9 ± 3.9	13.2 ± 13.6*	2.913	0.013	5.1 ± 8.4	0.618	0.872	7.3 ± 14.4	1.275	0.444
Post-implantation Loss (%)	0.9 ± 2.2	7.4 ± 14.2*	2.789	0.018	2.7 ± 3.4	0.779	0.778	0.8 ± 2.3	0.064	>0.999

All group compared with the negative control group, statistical significance was indicated as follows: **P<0.01, *P<0.05. n = X rats per group.

### 3.4 Embryo-fetal developmental toxicity

The mean body length of female fetuses in the high-dose group was significantly greater than that in the negative control group (*P* < 0.05). This finding was considered incidental and unrelated to the test article, and was likely attributable to inter-individual variation. No statistically significant differences in fetal sex ratio were observed among the high-dose, low-dose, adjuvant control, and negative control groups (*P*≥0.05) (**Table 2**).

**Table 2:** Fetal growth parameters and sex distribution.

Parameters	Negative control group (n=20)	Adjuvant control group (n=18)			Low dose group (n=20)			High dose group (n=20)		
			χ^2^	*P*		χ^2^	*P*		χ^2^	*P*
Fetal Weight(g)										
Male	4.0 ± 0.3	3.9 ± 0.3	0.911	0.692	4.0 ± 0.2	0.089	>0.999	3.9 ± 0.5	0.399	0.960
Female	3.8 ± 0.3	3.6 ± 0.4	1.570	0.280	3.7 ± 0.2	0.413	0.956	3.8 ± 0.2	0.767	0.786
Crown-Rump Length(nm)										
Male	35.3 ± 1.7	35.3 ± 1.6	0.015	>0.999	35.4 ± 2.4	0.129	0.999	36.0 ± 2.8	1.041	0.602
Female	34.2 ± 1.9	34.2 ± 1.7	0.086	>0.999	34.5 ± 2.3	0.416	0.955	35.7 ± 1.7*	2.470	0.042
sex ratio(M/F)	0.9(151:160)	1.0(125:124)	0.150	0.381	1.0(151:147)	0.273	0.329	0.9(141:158)	0.119	0.396

All group compared with the negative control group, statistical significance was indicated as follows: *P<0.05. n = X rats per group. M:Male; F:Female.

The adjuvant control group showed significantly higher litter-based and fetus-based frequencies of resorbed embryos and dead fetuses than the negative control group (*P* < 0.05), while the fetus-based frequency of live fetuses was significantly lower(*P* < 0.05). However, these changes were not consistently reflected across litter-based positive rates, and similar findings were not reproduced in the vaccine-treated groups. In addition, no clear dose-response relationship or associated embryo-fetal developmental abnormalities were observed. All indices of fetal classification in the high and low dose groups showed no statistically significant differences compared with the negative control group (*P*≥0.05). Therefore, these findings did not indicate a clear treatment-related toxicological effect under the conditions of this study (**Supplementary Table 6**).

### 3.5 Teratogenicity

#### 3.5.1 Examination of placentas and external fetal morphology

In the negative control group, 20 litters comprising 311 fetuses were examined. Placental abnormalities, specifically placental fusion, were observed in 1 of 20 litters and in 2 of 311 fetuses, corresponding to a litter-based positive rate of 0.6 ± 2.8%. External abnormalities, specifically thread-like tail, were observed in 1 of 20 litters and in 2 of 311 fetuses, corresponding to a litter-based positive rate of 0.3 ± 1.5%.

In the adjuvant control group, 18 litters comprising 250 fetuses were examined. Placental abnormalities, specifically placental fusion, were observed in 1 of 18 litters and in 2 of 250 fetuses, corresponding to a litter-based positive rate of 0.7 ± 2.8%. No external fetal abnormalities were observed.

In both the low-dose group (20 litters, 298 fetuses) and the high-dose group (20 litters, 299 fetuses), no placental abnormalities or external fetal abnormalities were detected.

#### 3.5.2 Fetal skeletal examination

##### 3.5.2.1 Ossification counts and degree of sternebral ossification

No statistically significant differences were observed in the number of ossified metacarpals, metatarsals, sternebrae, or total sacrocaudal vertebrae in the adjuvant control or high-dose group compared with the negative control group (*P*≥0.05).

The fetus-based frequency of an unossified fifth sternebra in the high-dose group was significantly lower than that in the negative control group (*P* < 0.05), indicating a higher degree of ossification in the high dose group. This finding was considered unrelated to the test article and without toxicological significance. No other significant abnormalities were noted (**Supplementary Table 7**).

##### 3.5.2.2 Skeletal abnormalities and variations

Skeletal variations observed across all groups included incomplete ossification of the parietal bones, interparietal bones, and supraoccipital bones; unossified or incompletely ossified hyoid bones; dumbbell ossification of thoracic vertebral centra; bilateral ossification of thoracic vertebral centra; incomplete ossification of sacral vertebral arches; lumbar ribs; incomplete ossification or dumbbell ossification of sternebrae; and incomplete ossification of the pubic bones.

In the negative control group, 20 litters comprising 159 fetuses were examined. In addition to the variations common to all groups, unilateral ossification of thoracic vertebral centra was also observed. The litter-based positive rate for skeletal variations was 66.4 ± 24.9%, occurring in 20/20 litters and affecting 106/159 fetuses. No skeletal malformations were observed.

In the adjuvant control group, 18 litters comprising 127 fetuses were examined. Variations specific to this group included wavy ribs and incomplete ossification of the ischial bones. The litter-based positive rate for skeletal variations was 68.8 ± 30.8%, occurring in 18/18 litters and affecting 84/127 fetuses. No skeletal malformations were detected. The fetus-based frequencies of incomplete ossification of the interparietal bones, supraoccipital bones, and sacral vertebral arches were significantly higher than those in the negative control group (*P* < 0.05). However, no significant differences were observed in litter-based positive rates or in the number of affected litters, indicating that these findings were likely attributable to litter effects rather than the test article. No other significant abnormalities were noted.

In the high-dose group, 20 litters comprising 155 fetuses were examined. Variations specific to this group included unilateral ossification of thoracic vertebral centra and incomplete ossification of the ischial bones. The litter-based positive rate for skeletal variations was 60.4 ± 27.5%, occurring in 20/20 litters and affecting 94/155 fetuses. No skeletal malformations were observed. The fetus-based frequency of incomplete ossification of the interparietal bones was significantly higher than that in the negative control group (*P* < 0.05), but no significant differences were found in litter-based positive rates or in the number of affected litters, suggesting that this finding was attributable to a litter effect and was unrelated to the test article. No other significant abnormalities were noted (**Supplementary Table 8**).

#### 3.5.3 Visceral examination of fetuses

In the negative control group, 20 litters comprising 152 fetuses were examined. Visceral variations observed included thymic horns, renal pelvic dilation, and ureteral tortuosity. The litter-based positive rate for visceral variations was 10.7 ± 14.1%, occurring in 9/20 litters and affecting 16/152 fetuses. No visceral malformations were detected.

In the adjuvant control group, 18 litters comprising 122 fetuses were examined. Visceral variations included transposition of umbilical arteries, thymic horns, and renal pelvic dilation. The litter-based positive rate for visceral variations was 19.7 ± 25.2%, occurring in 10/18 litters and affecting 18/122 fetuses. No visceral malformations were observed.

In the high-dose group, 20 litters comprising 144 fetuses were examined. Visceral variations included transposition of umbilical arteries, thymic horns, renal pelvic dilation, and ureteral tortuosity. The litter-based positive rate for visceral variations was 11.3 ± 9.8%, occurring in 13/20 litters and affecting 17/144 fetuses. No visceral malformations were noted (**Supplementary Table 9**).

### 3.6 Immunogenicity assessment

**Figure 6:**
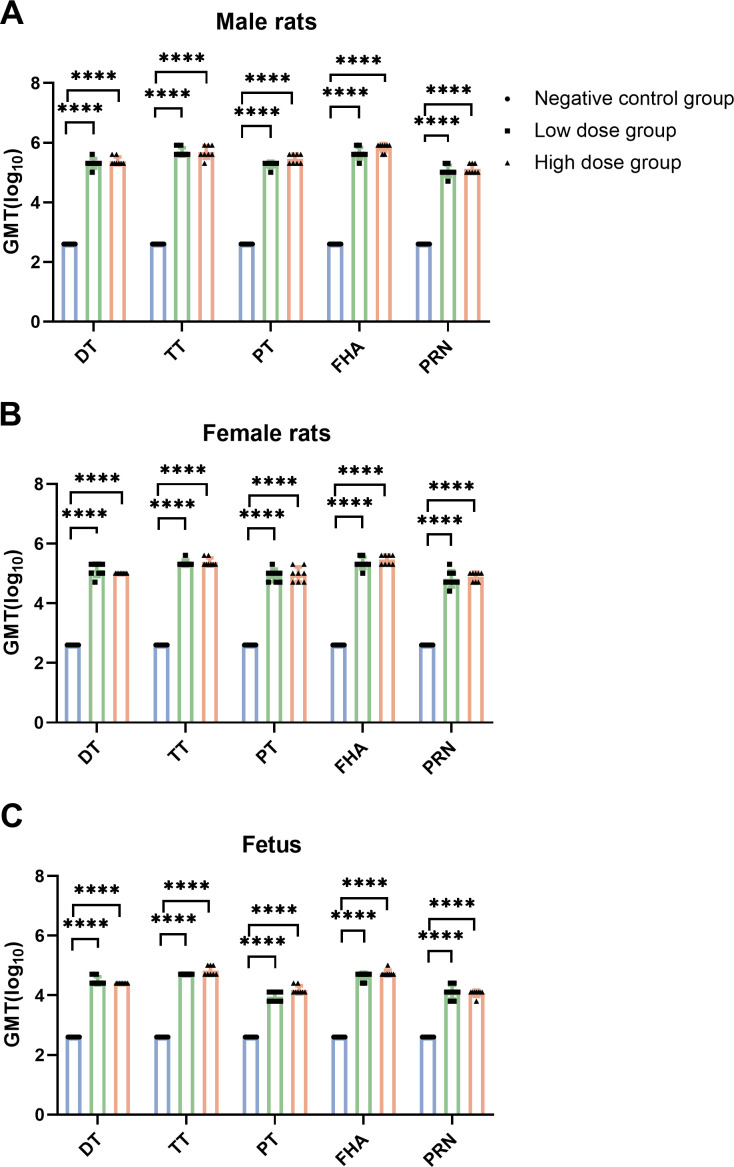
Antibody titers against pertussis, diphtheria, and tetanus components in three groups. Statistical significance was determined by two-way ANOVA followed by Dunnett’s multiple comparisons test. **(A)** male rats. **(B)** female rats. **(C)** fetus. Antibody titers in all vaccine-treated groups (low- and high-dose) were significantly higher than those in the negative control group. Statistical significance was indicated as follows: ****P<0.0001.

All anti-pertussis, anti-diphtheria, and anti-tetanus antibody titers were negative in male rats, female rats, and fetuses from the negative control group (**Figure 6**).

In the high-dose and low-dose male rat groups, antibody titers against all vaccine components (DT, TT, PT, FHA, and PRN) were positive and were significantly different from those in the negative control group (*P* < 0.01). Antibody levels against DT, PT, FHA, and PRN increased in a dose-dependent manner, whereas anti-TT titers were comparable between the low- and high-dose groups.

In the high- and low-dose female rat groups, antibody titers against all components were positive and significantly different from those in the negative control group (*P* < 0.01). Titers against TT, PT, FHA, and PRN exhibited dose-dependent increases, whereas anti-DT titers remained largely comparable across dose groups.

In fetuses from the high- and low-dose groups, antibody titers against all components were positive and significantly higher than those in the negative control group (*P* < 0.01). A dose-dependent increase was observed for anti-TT, anti-PT, and anti-FHA titers, whereas anti-DT and anti-PRN titers remained generally comparable between the two dose groups. The presence of antigen-specific IgG antibodies in fetal serum indicates efficient transplacental transfer of maternal antibodies.

## 4 Discussion

Currently, pertussis remains one of the least well-controlled vaccine-preventable diseases, posing a particularly serious threat to infants younger than one year of age (12). Epidemiological data indicate that this age group accounts for the majority of pertussis-related deaths (13), with the highest risk observed in infants younger than three months (14). In response to the increasing burden of disease among young infants, maternal immunization has been adopted in several countries as an effective preventive strategy (15–17). Vaccination during pregnancy not only protects the mother but also enables the transplacental transfer of antibodies to the fetus, thereby providing passive protection to newborns during early life, when they are most vulnerable to severe disease (18).

Maternal antibodies have been shown to confer protection against Bordetella pertussis infection in early infancy without interfering with subsequent immune responses following primary immunization (15, 19–21). At the same time, adolescents and adults with waning immunity have been identified as important sources of transmission to infants (22). Because pertussis infection in these populations often presents with atypical symptoms, it may go unrecognized, facilitating ongoing transmission within households and communities (23). Strategies such as cocooning, which involves vaccinating close contacts of newborns, have therefore been proposed as complementary approaches to reduce infant exposure (24, 25).

The use of vaccines in adults, particularly in women of childbearing age, requires careful evaluation of potential reproductive and developmental toxicity. This is especially important for adjuvanted vaccines administered during pregnancy or the periconceptional period, where potential effects on fertility, embryo implantation, and fetal development must be systematically assessed.

In the present study, no evidence of reproductive toxicity or embryo-fetal developmental toxicity was observed following repeated intramuscular administration of the adsorbed acellular diphtheria-tetanus-pertussis (reduced-dose) combined vaccine in male and female rats, including pregnant animals. No adverse effects were detected in general clinical observations, body weight, food consumption, or reproductive performance parameters, such as the number of corpora lutea and implantation sites. Furthermore, no treatment-related effects were observed on fetal survival, external morphology, visceral development, or skeletal formation, indicating the absence of embryo-fetal developmental toxicity or teratogenicity under the conditions of this study.

Importantly, although several reproductive and embryo-fetal parameters in the adjuvant control group differed statistically from those in the negative control group, these findings were not reproduced in the vaccine-treated groups and were not accompanied by a consistent dose-response pattern, maternal toxicity, fetal malformations, or other developmental abnormalities. Therefore, the overall data did not indicate a clear treatment-related toxicological effect of the aluminum-based adjuvant under the conditions of this study.

Immunogenicity assessments demonstrated that vaccination induced robust antigen-specific IgG responses in maternal animals, and substantial levels of maternally derived antibodies were detected in fetal serum. These findings confirm efficient transplacental antibody transfer and suggest the potential for passive protection of offspring. Taken together, the absence of reproductive toxicity, combined with the induction of robust maternal and fetal antibody responses, supports a favorable benefit-risk profile for this vaccine in the context of maternal immunization.

Several acellular pertussis-containing vaccines for adolescents and adults, such as such as Boostrix (GlaxoSmithKline) and Adacel (Sanofi) (26, 27), have been widely used internationally and have demonstrated favorable safety and tolerability profiles, including in pregnant women. These vaccines are typically formulated with aluminum-based adjuvants and produced using component-based purification strategies. Similar to these licensed vaccines, the Tdacp vaccine evaluated in this study is an aluminum-adjuvanted, component acellular pertussis vaccine. The use of column chromatography-based purification enables the production of well-defined antigen components with high purity and controlled antigen content, which may contribute to improved manufacturing consistency and quality control.

Although such vaccines have been widely implemented in many countries, no comparable pertussis-containing vaccines intended for adolescents, adults, or pregnant women have yet been approved in China. This highlights an important unmet need and supports the development of vaccines suitable for booster and maternal immunization strategies in the Chinese population.

In conclusion, the Tdacp vaccine demonstrated no adverse effects on fertility, early embryonic development, or embryo-fetal development in rats, while eliciting robust immune responses. These findings provide important nonclinical safety data and support the continued development and clinical evaluation of this vaccine for booster immunization in adolescents and adults, as well as for potential use in maternal immunization programs.

A limitation of this study is that immunological assessment was restricted to antigen-specific antibody responses in vaccine-treated animals, and the adjuvant control group was not included in the antibody analysis. Although antigen-specific IgG responses were not expected in this group because no vaccine antigens were administered, its inclusion could have provided additional information regarding baseline assay signal, assay specificity, and potential non-specific immune activation. In addition, cytokine profiling and early post-dose inflammatory markers were not evaluated in the present study. These endpoints are being investigated in separate immunogenicity studies and were therefore beyond the scope of the present reproductive toxicity study. Future studies should consider incorporating broader immunological assessments, including the adjuvant control group, particularly when biological changes are observed in adjuvant-treated animals.

## Data Availability

The original contributions presented in the study are included in the article/**Supplementary Material**. Further inquiries can be directed to the corresponding authors.
