# Supplementary material for: Preclinical study on an adsorbed acellular diphtheria- tetanus-pertussis (reduced-dose) combined vaccine: evaluation of reproductive safety and immunogenicity
Source: Front Immunol. 2026 May 26;17:1828345. doi: 10.3389/fimmu.2026.1828345 (PMC13246618; doi:10.3389/fimmu.2026.1828345)
Supplement: Supplementary file 1 [file Table1.docx]

Supplementary Material

# Supplementary Tables

## Supplementary table 1. Statistical data of food intake of experimental animals(MEAN±SD, g/d/rat)

| Time of measurement | | | Negative control group | Adjuvant control group | | | Low dose group | | | High dose group | | |
| --- | --- | --- | --- | --- | --- | --- | --- | --- | --- | --- | --- | --- |
|  |  |  |  |  | χ^2^ | *P* |  | χ^2^ | *P* |  | χ^2^ | *P* |
| Male | n |  | 20 | 20 |  |  | 20 |  |  | 20 |  |  |
|  | Pre-mating period | Week 1(First immunization) | 26.1±1.7 | 27.8±1.3 | 1.958 | 0.146 | 27.7±2.4 | 1.841 | 0.182 | 28.0±1.0 | 2.253 | 0.082 |
|  |  | Week 2 | 27.2±1.5 | 28.3±2.9 | 1.046 | 0.599 | 29.2±1.9 | 1.902 | 0.362 | 28.7±1.9 | 1.426 | 0.362 |
|  |  | Week 3(Second immunization) | 25.8±1.6 | 26.5±2.6 | 0.739 | 0.802 | 25.2±1.6 | 0.713 | 0.818 | 25.8±1.5 | ＜0.001 | ＞0.999 |
| Female | n |  | 20 | 20 |  |  | 20 |  |  | 20 |  |  |
|  | Pre-mating period | Week 1 | 17.6±2.5 | 20.0±0.9 | 1.879 | 0.170 | 18.0±3.5 | 0.261 | 0.988 | 19.8±2.3 | 1.768 | 0.207 |
|  |  | Week 2 | 20.7±2.2 | 19.5±2.0 | 1.176 | 0.512 | 18.5±1.5 | 2.288 | 0.076 | 19.4±2.0 | 1.293 | 0.439 |
|  |  | Week 3(First immunization) | 17.5±2.4 | 17.9±2.5 | 0.346 | 0.972 | 18.3±2.7 | 0.714 | 0.817 | 16.5±1.4 | 0.866 | 0.721 |
|  | n |  | 20 | 18 |  |  | 20 |  |  | 20 |  |  |
|  | Gestation period | GD0-GD3 | 22.0±2.7 | 22.2±2.0 | 0.271 | 0.987 | 22.0±2.4 | 0.021 | ＞0.999 | 22.2±2.0 | 0.218 | 0.993 |
|  |  | GD3-GD6(Second immunization) | 24.9±2.0 | 25.0±1.8 | 0.146 | 0.999 | 24.7±3.1 | 0.284 | 0.985 | 25.1±2.5 | 0.290 | 0.984 |
|  |  | GD6-GD9 | 24.5±3.2 | 26.3±3.6 | 1.880 | 0.157 | 25.3±2.8 | 0.908 | 0.693 | 24.8±2.5 | 0.379 | 0.965 |
|  |  | GD9-GD12 | 26.3±2.8 | 26.5±2.4 | 0.275 | 0.986 | 25.8±3.2 | 0.574 | 0.893 | 26.0±2.0 | 0.281 | 0.985 |
|  |  | GD12-GD15 | 26.6±3.0 | 26.8±2.6 | 0.212 | 0.993 | 25.6±3.3 | 1.093 | 0.564 | 26.3±2.3 | 0.323 | 0.978 |
|  |  | GD15-GD18 | 27.9±2.6 | 28.3±2.4 | 0.386 | 0.963 | 27.4±2.8 | 0.665 | 0.847 | 27.8±2.0 | 0.217 | 0.993 |
|  |  | GD18-GD20 | 28.1±2.4 | 28.6±2.9 | 0.573 | 0.894 | 27.3±2.7 | 1.024 | 0.612 | 27.3±2.1 | 1.061 | 0.586 |

## Supplementary table 2. Statistical data of body weight of experimental animals(MEAN±SD, g/d/rat)

| Time of measurement | | | Negative control group | Adjuvant control group | | | Low dose group | | | High dose group | | |
| --- | --- | --- | --- | --- | --- | --- | --- | --- | --- | --- | --- | --- |
|  |  |  |  |  | χ^2^ | *P* |  | χ^2^ | *P* |  | χ^2^ | *P* |
| Male | n |  | 20 | 20 |  |  | 20 |  |  | 20 |  |  |
|  | Quarantine period | Week 0 | 378±14 | 377±13 | 0.144 | 0.998 | 377±12 | 0.299 | 0.982 | 377±12 | 0.224 | 0.992 |
|  | Pre-mating period | Week 0.5 | 393±13 | 397±16 | 0.683 | 0.835 | 395±20 | 0.314 | 0.979 | 393±14 | 0.014 | ＞0.999 |
|  |  | Week 1(First immunization) | 404±14 | 407±17 | 0.502 | 0.924 | 407±20 | 0.552 | 0.903 | 408±15 | 0.618 | 0.870 |
|  |  | Week 1.5 | 422±15 | 426±21 | 0.699 | 0.826 | 428±23 | 1.109 | 0.551 | 428±17 | 1.040 | 0.598 |
|  |  | Week 2 | 432±15 | 436±24 | 0.606 | 0.877 | 442±25 | 1.388 | 0.374 | 438±19 | 0.884 | 0.707 |
|  |  | Week 2.5 | 443±19 | 447±26 | 0.453 | 0.942 | 453±27 | 1.303 | 0.424 | 447±22 | 0.431 | 0.950 |
|  |  | Week 3(Second immunization) | 451±19 | 454±28 | 0.414 | 0.955 | 462±27 | 1.409 | 0.362 | 455±23 | 0.526 | 0.914 |
| Female | n |  | 20 | 18 |  |  | 20 |  |  | 20 |  |  |
|  | Quarantine period | Week 0 | 241±10 | 241±11 | 0.005 | ＞0.999 | 242±10 | 0.078 | ＞0.999 | 242±10 | 0.012 | ＞0.999 |
|  | Pre-mating period | Week 0.5 | 249±12 | 250±12 | 0.243 | 0.990 | 248±10 | 0.428 | 0.951 | 248±12 | 0.317 | 0.979 |
|  |  | Week 1 | 254±14 | 256±13 | 0.512 | 0.920 | 252±11 | 0.689 | 0.831 | 253±13 | 0.360 | 0.969 |
|  |  | Week 1.5 | 264±15 | 266±14 | 0.372 | 0.966 | 259±13 | 1.157 | 0.519 | 261±14 | 0.725 | 0.810 |
|  |  | Week 2 | 269±16 | 269±14 | 0.006 | ＞0.999 | 262±13 | 1.566 | 0.280 | 266±15 | 0.564 | 0.892 |
|  |  | Week 2.5 | 274±18 | 276±16 | 0.376 | 0.965 | 267±13 | 1.420 | 0.355 | 270±16 | 0.788 | 0.771 |
|  |  | Week 3(First immunization) | 277±19 | 279±16 | 0.449 | 0.944 | 268±14 | 1.812 | 0.179 | 272±17 | 0.965 | 0.651 |
|  | n |  | 20 | 18 |  |  | 20 |  |  | 20 |  |  |
|  | Gestation period | GD0 | 283±19 | 286±19 | 0.541 | 0.908 | 277±16 | 1.065 | 0.584 | 280±17 | 0.677 | 0.840 |
|  |  | GD3 | 304±20 | 308±19 | 0.768 | 0.785 | 297±17 | 1.160 | 0.519 | 298±18 | 0.929 | 0.678 |
|  |  | GD6(Second immunization) | 318±20 | 320±20 | 0.279 | 0.985 | 312±20 | 0.988 | 0.637 | 313±19 | 0.841 | 0.738 |
|  |  | GD9 | 330±22 | 333±22 | 0.435 | 0.949 | 327±22 | 0.522 | 0.916 | 326±19 | 0.671 | 0.843 |
|  |  | GD12 | 349±22 | 351±24 | 0.314 | 0.979 | 342±25 | 0.927 | 0.679 | 341±20 | 1.103 | 0.558 |
|  |  | GD15 | 368±24 | 369±25 | 0.054 | ＞0.999 | 362±26 | 0.769 | 0.784 | 361±22 | 1.003 | 0.627 |
|  |  | GD18 | 413±24 | 407±29 | 0.637 | 0.862 | 404±29 | 1.005 | 0.626 | 403±27 | 1.196 | 0.495 |
|  |  | GD20 | 447±28 | 438±35 | 0.927 | 0.679 | 438±30 | 0.923 | 0.669 | 435±31 | 1.232 | 0.471 |

## Supplementary table 3. Statistical data of Animal Visceral Organ Wet Weights(MEAN±SD)

| Parameters | | | Negative control group | Adjuvant control group | | | Low dose group | | | High dose group | | |
| --- | --- | --- | --- | --- | --- | --- | --- | --- | --- | --- | --- | --- |
|  |  |  |  |  | χ^2^ | *P* |  | χ^2^ | *P* |  | χ^2^ | *P* |
| Male | n |  | 20 | 20 |  |  | 20 |  |  | 20 |  |  |
|  | Testis(L) | Organ wet weight(g) | 1.7867±0.1318 | 1.7193±0.1770 | 1.369 | 0.385 | 1.7377±0.1683 | 0.996 | 0.630 | 1.7015±0.1416 | 1.731 | 0.209 |
|  |  | Organ Coefficient  (g/100g) | 0.3549±0.0267 | 0.3385±0.0330 | 1.620 | 0.255 | 0.3397±0.0378 | 1.504 | 0.311 | 0.3354±0.0293 | 1.924 | 0.143 |
|  | Testis(R) | Organ wet weight(g) | 1.7730±0.1391 | 1.7317±0.1806 | 0.801 | 0.762 | 1.7502±0.1853 | 0.442 | 0.946 | 1.7272±0.1412 | 0.889 | 0.704 |
|  |  | Organ Coefficient  (g/100g) | 0.3520±0.0258 | 0.3409±0.0332 | 1.061 | 0.584 | 0.3421±0.0404 | 0.949 | 0.662 | 0.3406±0.0304 | 1.098 | 0.558 |
|  | Epididymis(L) | Organ wet weight(g) | 0.6696±0.0545 | 0.6382±0.0407 | 2.098 | 0.099 | 0.6584±0.0542 | 0.749 | 0.793 | 0.6314±0.0370* | 2.551 | 0.034 |
|  |  | Organ Coefficient  (g/100g) | 0.1331±0.0119 | 0.1257±0.0090 | 1.826 | 0.174 | 0.1290±0.0157 | 1.021 | 0.612 | 0.1249±0.0131 | 2.049 | 0.110 |
|  | Epididymis(R) | Organ wet weight(g) | 0.6720±0.0539 | 0.6566±0.0529 | 0.912 | 0.688 | 0.6652±0.0591 | 0.399 | 0.959 | 0.6554±0.0467 | 0.984 | 0.637 |
|  |  | Organ Coefficient  (g/100g) | 0.1336±0.0126 | 0.1295±0.0125 | 0.989 | 0.634 | 0.1300±0.0129 | 0.871 | 0.716 | 0.1296±0.0143 | 0.978 | 0.642 |
| Female | n |  | 20 | 18 |  |  | 20 |  |  | 20 |  |  |
|  | Ovary(L) | Organ wet weight(g) | 0.1152±0.1833 | 0.0703±0.0132 | 1.470 | 0.330 | 0.0690±0.0184 | 1.552 | 0.288 | 0.0651±0.0191 | 1.685 | 0.228 |
|  |  | Organ Coefficient(g/100g) | 0.0264±0.0439 | 0.0160±0.0023 | 1.423 | 0.356 | 0.0158±0.0043 | 1.482 | 0.324 | 0.0149±0.0041 | 1.611 | 0.260 |
|  | Ovary(R) | Organ wet weight(g) | 0.0743±0.0159 | 0.0696±0.0221 | 0.735 | 0.806 | 0.0746±0.0250 | 0.055 | ＞0.999 | 0.0681±0.0128 | 1.006 | 0.624 |
|  |  | Organ Coefficient(g/100g) | 0.0167±0.0038 | 0.0158±0.0043 | 0.626 | 0.868 | 0.0172±0.0058 | 0.344 | 0.973 | 0.0158±0.0034 | 0.652 | 0.854 |
|  | Litter Weight(g) |  | 89.8±6.4 | 79.1±25.8 | 1.996 | 0.124 | 86.6±10.2 | 0.618 | 0.872 | 88.1±17.7 | 0.335 | 0.975 |

## Supplementary table 4. Sperm count, motility, and morphology in the epididymis of male rats(MEAN±SD)

| Parameters | Negative control group (n=20) | Adjuvant control group (n=18) | | | Low dose group (n=20) | | | High dose group (n=20) | | |
| --- | --- | --- | --- | --- | --- | --- | --- | --- | --- | --- |
|  |  |  | χ^2^ | *P* |  | χ^2^ | *P* |  | χ^2^ | *P* |
| Sperm Motility(%) | 47.1±12.4 | 47.3±11.4 | 0.065 | 0.999 | 39.7±10.0 | 1.917 | 0.145 | 44.9±14.2 | 0.559 | 0.899 |
| Epididymal Sperm Count(×106/g) | 447.7±101.2 | 477.1±154.5 | 0.664 | 0.846 | 446.9±182.9 | 0.018 | ＞0.999 | 495.8±105.2 | 1.086 | 0.567 |
| Sperm Morphology(%) | 6.3±3.6 | 6.0±3.6 | 0.380 | 0.964 | 5.0±2.2 | 1.466 | 0.331 | 6.2±1.6 | 0.081 | 0.999 |

## Supplementary table 5. Vaginal cytology (estrous cycle smear) analysis in female rats(MEAN±SD)

| Parameters | Negative control group (n=20) | Adjuvant control group (n=18) | | | Low dose group (n=20) | | | High dose group (n=20) | | |
| --- | --- | --- | --- | --- | --- | --- | --- | --- | --- | --- |
|  |  |  | χ^2^ | *P* |  | χ^2^ | *P* |  | χ^2^ | *P* |
| Estrous Cycle Length (days) | 4.0±0 | 4.1±0.4 | 1.385 | 0.375 | 4.0±0.4 | 0.422 | 0.953 | 4.0±0.1 | 0.181 | 0.996 |

## Supplementary table 6. Classification and incidence of fetal deaths and malformations

| Parameters | Negative control group | | |  | Adjuvant control group | | | | | | | | |  | Low dose group | | | | | | | | |  | High dose group | | | | | | | | |
| --- | --- | --- | --- | --- | --- | --- | --- | --- | --- | --- | --- | --- | --- | --- | --- | --- | --- | --- | --- | --- | --- | --- | --- | --- | --- | --- | --- | --- | --- | --- | --- | --- | --- |
|  | Incidence  (% of Litters) | Affected Litters(No. Positive/Total) | Affected Fetuses  (No. Positive/Total) |  | Incidence (% of Litters) | | | Affected Litters (No. Positive/Total) | | | Affected Fetuses (No. Positive/Total) | | |  | Incidence (% of Litters) | | | Affected Litters (No. Positive/Total) | | | Affected Fetuses (No. Positive/Total) | | |  | Incidence (% of Litters) | | | Affected Litters(No. Positive/Total) | | | Affected Fetuses (No. Positive/Total) | | |
|  |  |  |  |  |  | χ^2^ | *P* |  | χ^2^ | *P* |  | χ^2^ | *P* |  |  | χ^2^ | *P* |  | χ^2^ | *P* |  | χ^2^ | *P* |  |  | χ^2^ | *P* |  | χ^2^ | *P* |  | χ^2^ | *P* |
| Live Fetuses | 99.1±2.2 | 20/20 | 311/314 |  | 92.6±14.2* | 2.786 | 0.019 | 18/18 | / | / | 249/263** | 9.549 | 0.002 |  | 97.3±3.4 | 0.775 | 0.781 | 20/20 | / | / | 298/306 | 2.447 | 0.103 |  | 99.3±2.3 | 0.064 | ＞0.999 | 20/20 | / | / | 299/301 | 0.161 | 0.520 |
| Implantation Scar | 0 | 0/20 | 0/314 |  | 0 | / | / | 0/18 | / | / | 0/263 | / | / |  | 0 | / | / | 0/20 | / | / | 0/306 | / | / |  | 0 | / | / | 0/20 | / | / | 0/301 | / | / |
| Resorptions | 0.6±1.9 | 2/20 | 2/314 |  | 5.2±9.8* | 2.769 | 0.020 | 7/18* | 4.374 | 0.043 | 10/263** | 7.042 | 0.008 |  | 1.4±2.9 | 0.484 | 41/44 | 4/20 | 0.784 | 32/97 | 4/306* | 0.726 | 0.331 |  | 0.8±2.3 | 0.092 | 1 | 2/20 | / | / | 2/301 |  |  |
| Placental Remnant | 0.3±1.3 | 1/20 | 1/314 |  | 0.7±2.2 | 0.875 | 0.715 | 2/18 | 0.487 | 0.459 | 2/263 | 0.541 | 0.434 |  | 0.6±1.9 | 0.670 | 0.844 | 2/20 | 0.360 | 0.500 | 2/306 | 0.361 | 0.490 |  | 0 | 0.590 | 0.886 | 0/20 | / | / | 0/301 | / | / |
| Early Macerated Fetus | 0 | 0/20 | 0/314 |  | 1.1±4.7 | 1.443 | 32/93 | 1/18 | 1.141 | 0.474 | 1/263 | 1.196 | 0.456 |  | 0.3±1.4 | 0.420 | 81/85 | 1/20 | 1.026 | 0.500 | 1/306 | 1.028 | 0.494 |  | 0 | ＜0.001 | ＞0.999 | 0/20 | / | / | 0/301 | / | / |
| Late Macerated Fetus | 0 | 0/20 | 0/314 |  | 0 | ＜0.001 | ＞0.999 | 0/18 | / | / | 0/263 | / | / |  | 0.3±1.5 | 1.395 | 0.371 | 1/20 | 1.026 | 0.500 | 1/306 | 1.028 | 0.494 |  | 0 | ＜0.001 | ＞0.999 | 0/20 | / | / | 0/301 | / | / |
| Dead Fetus | 0 | 0/20 | 0/314 |  | 0.3±1.4 | 1.514 | 27/88 | 1/18 | 1.141 | 0.474 | 1/263 | 1.196 | 0.456 |  | 0 | ＜0.001 | ＞0.999 | 0/20 | / | / | 0/304 | / | / |  | 0 | ＜0.001 | ＞0.999 | 0/20 | / | / | 0/301 | / | / |
| Dead Embryo | 0.9±2.2 | 3/20 | 3/314 |  | 7.4±14.2* | 2.789 | 0.018 | 9/18* | 5.371 | 0.024 | 14/263** | 9.549 | 0.002 |  | 2.7±3.4 | 0.779 | 0.778 | 8/20 | 3.315 | 0.078 | 8/304 | 2.482 | 0.101 |  | 0.8±2.3 | 0.064 | ＞0.999 | 2/20 | 0.229 | 0.500 | 2/301 | 0.161 | 0.520 |

All group compared with the negative control group, data are presented as mean ± SD. Statistical significance was indicated as follows: ***P*＜0.01, **P*＜0.05. n = X rats per group.

## Supplementary table 7. Counts of ossification centers in the sternum and appendicular skeleton of rat fetuses

| Parameters | Negative control group | | |  | Adjuvant control group | | | | | | | | |  | High dose group | | | | | | | | |
| --- | --- | --- | --- | --- | --- | --- | --- | --- | --- | --- | --- | --- | --- | --- | --- | --- | --- | --- | --- | --- | --- | --- | --- |
|  |  |  |  |  |  |  |  | χ^2^ | | | *P* | | |  |  |  |  | χ^2^ | | | *P* | | |
| Number of Ossified Metacarpals | 7.7±0.4 | | |  | 7.4±0.8 | | | 1.645 | | | 0.185 | | |  | 7.7±0.4 | | | 0.118 | | | 0.990 | | |
| Number of Ossified Metatarsals | 8.0±0.0 | | |  | 8.0±0.0 | | | 0.166 | | | 0.980 | | |  | 8.0±0.2 | | | 1.021 | | | 0.495 | | |
| Number of Ossified Sternobrae | 5.6±0.5 | | |  | 5.7±0.4 | | | 0.396 | | | 0.894 | | |  | 5.7±0.5 | | | 0.437 | | | 0.872 | | |
| Total Number of Ossified Sacrocaudal Vertebrae | 8.0±0.4 | | |  | 8.0±0.5 | | | 0.072 | | | 0.996 | | |  | 8.0±0.4 | | | 0.557 | | | 0.802 | | |
|  | Incidence (% of Litters) | Affected Litters (No. Positive/Total) | Affected Fetuses(No. Positive/Total) |  | Incidence (% of Litters) | | | Affected Litters(No. Positive/Total) | | | Affected Fetuses (No. Positive/Total) | | |  | Incidence (% of Litters) | | | Affected Litters (No. Positive/Total) | | | Affected Fetuses  (No. Positive/Total) | | |
|  |  |  |  |  |  | χ^2^ | *P* |  | χ^2^ | *P* |  | χ^2^ | *P* |  |  | χ^2^ | *P* |  | χ^2^ | *P* |  | χ^2^ | *P* |
| Unossified Sternebra 1 | 1.3±3.8 | 2/20 | 2/159 |  | 0 | 1.221 | 0.375 | 0/18 | 1.900 | 0.270 | 0/127 | 1.609 | 0.308 |  | 0.8±3.7 | 0.416 | 0.883 | 1/20 | 0.360 | 0.500 | 1/155 | 0.311 | 0.510 |
| Unossified Sternebra 2 | 1.3±3.8 | 2/20 | 2/159 |  | 1.6±4.6 | 0.159 | 0.982 | 2/18 | 0.012 | 0.656 | 2/127 | 0.051 | 0.600 |  | 3.8±9.5 | 1.203 | 0.385 | 3/20 | 0.229 | 0.500 | 4/155 | 0.733 | 0.330 |
| Unossified Sternebra 3 | 0.6±2.8 | 1/20 | 1/159 |  | 0 | 0.380 | 0.901 | 0/18 | 0.924 | 0.526 | 0/127 | 0.802 | 0.556 |  | 2.5±8.2 | 1.171 | 0.403 | 2/20 | 0.360 | 0.500 | 2/155 | 0.363 | 0.490 |
| Unossified Sternebra 4 | 0.6±2.8 | 1/20 | 1/159 |  | 0.8±3.4 | 0.157 | 0.982 | 1/18 | 0.006 | 0.730 | 1/127 | 0.026 | 0.692 |  | 0.8±3.7 | 0.200 | 0.971 | 1/20 | / | / | 1/155 | ＜0.001 | 0.744 |
| Unossified Sternebra 5 | 17.5±25.4 | 11/20 | 28/159 |  | 15.3±17.6 | 0.329 | 0.925 | 10/18 | 0.001 | 0.615 | 20/127 | 0.175 | 0.399 |  | 10.9±15.5 | 1.044 | 0.480 | 9/20 | 0.400 | 0.376 | 15/155* | 4.179 | 0.029 |
| Unossified Sternebra 6 | 16.0±23.3 | 9/20 | 26/159 |  | 12.3±25.3 | 0.539 | 0.813 | 6/18 | 0.540 | 0.345 | 16/127 | 0.794 | 0.236 |  | 10.9±14.4 | 0.768 | 0.664 | 10/20 | 0.100 | 0.500 | 15/155 | 3.080 | 0.056 |

All group compared with the negative control group, data are presented as mean ± SD. Statistical significance was indicated as follows: **P*＜0.01.

## Supplementary table 8. Incidence of skeletal malformations and variations in rat fetuses

| Parameters | Negative control group | | |  | Adjuvant control group | | | | |  | High dose group | | | |  |
| --- | --- | --- | --- | --- | --- | --- | --- | --- | --- | --- | --- | --- | --- | --- | --- |
|  | Incidence (% of Litters) | Affected Litters(No. Positive/Total) | Affected Fetuses(No. Positive/Total) |  | Incidence (% of Litters) | Affected Litters(No. Positive/Total) | Affected Fetuses (No. Positive/Total) | | |  | Incidence (% of Litters) | Affected Litters(No. Positive/Total) | Affected Fetuses  (No. Positive/Total) | | |
|  |  |  |  |  |  |  |  | χ^2^ | *P* |  |  |  |  | χ^2^ | *P* |
| Incomplete Ossification of Parietal | 6.1±10.9 | 6/20 | 10/159 |  | 9.4±14.8 | 8/18 | 11/127 | 0.584 | 0.295 |  | 8.1±14.0 | 6/20 | 11/155 | 0.082 | 0.476 |
| Incomplete Ossification of Interparietal | 20.2±26.9 | 13/20 | 33/159 |  | 32.7±26.0 | 16/18 | 41/127* | 4.893 | 0.019 |  | 30.1±25.3 | 15/20 | 46/155* | 3.319 | 0.045 |
| Incomplete Ossification of Supraoccipital | 6.7±12.3 | 6/20 | 11/159 |  | 13.9±23.5 | 8/18 | 18/127* | 4.079 | 0.034 |  | 8.7±17.0 | 6/20 | 11/155 | 0.004 | 0.563 |
| Unossified Hyoid | 14.5±18.0 | 11/20 | 24/159 |  | 14.3±22.7 | 7/18 | 19/127 | 0.001 | 0.555 |  | 13.6±19.9 | 9/20 | 19/155 | 0.822 | 0.229 |
| Incomplete Ossification of Hyoid | 16.7±16.7 | 12/20 | 27/159 |  | 11.8±13.9 | 10/18 | 15/127 | 1.506 | 0.145 |  | 15.5±17.1 | 12/20 | 24/155 | 0.129 | 0.418 |
| Dumbbell Ossification of Thoracic Centrum | 4.6±7.6 | 6/20 | 7/159 |  | 3.8±6.5 | 5/18 | 6/127 | 0.017 | 0.558 |  | 2.8±8.0 | 3/20 | 3/155 | 1.549 | 0.179 |
| Bilateral Ossification of Thoracic Centrum | 0.6±2.5 | 1/20 | 1/159 |  | 1.4±5.9 | 1/18 | 1/127 | 0.026 | 0.692 |  | 2.9±8.1 | 3/20 | 3/155 | 1.065 | 0.302 |
| Unilateral Ossification of Thoracic Centrum | 0.6±2.8 | 1/20 | 1/159 |  | 0 | 0/18 | 0/127 | 0.802 | 0.556 |  | 1.7±7.5 | 1/20 | 1/155 | ＜0.001 | 0.744 |
| Unilateral Ossification of Lumbar Centrum | 0 | 0/20 | 0/159 |  | 0 | 0/18 | 0/127 | / | / |  | 1.7±7.5 | 1/20 | 1/155 | 1.029 | 0.494 |
| Incomplete Ossification of Sacral Arch | 3.3±7.5 | 4/20 | 5/159 |  | 9.7±20.2 | 5/18 | 13/127* | 6.021 | 0.013 |  | 7.3±18.4 | 3/20 | 9/155 | 1.305 | 0.193 |
| Lumbar Rib | 1.9±6.1 | 2/20 | 3/159 |  | 0.8±3.4 | 1/18 | 1/127 | 0.619 | 0.400 |  | 3.6±8.4 | 4/20 | 4/155 | 0.173 | 0.486 |
| Wavy Rib | 0 | 0/20 | 0/159 |  | 2.8±11.8 | 1/18 | 2/127 | 2.522 | 0.196 |  | 0 | 0/20 | 0/155 | / | / |
| Incomplete Ossification of Sternebra | 36.5±24.0 | 19/20 | 57/159 |  | 42.7±29.4 | 16/18 | 50/127 | 0.374 | 0.312 |  | 26.4±22.1 | 16/20 | 42/155 | 2.785 | 0.061 |
| Dumbbell Ossification of Sternebra | 0.6±2.5 | 1/20 | 1/159 |  | 0.8±3.4 | 1/18 | 1/127 | 0.026 | 0.692 |  | 1.3±3.9 | 2/20 | 2/155 | 0.360 | 0.490 |
| Incomplete Ossification of Ischium | 0 | 0/20 | 0/159 |  | 0.8±3.4 | 1/18 | 1/127 | 1.256 | 0.444 |  | 1.3±5.6 | 1/20 | 2/155 | 2.065 | 0.243 |
| Incomplete Ossification of Pubis | 1.2±3.6 | 2/20 | 2/159 |  | 4.4±10.7 | 4/18 | 6/127 | 3.121 | 0.080 |  | 3.3±11.6 | 2/20 | 5/155 | 1.395 | 0.214 |
| Skeletal Variations | 66.4±24.9 | 20/20 | 106/159 |  | 68.8±30.8 | 18/18 | 84/127 | 0.009 | 0.512 |  | 60.4±27.5 | 20/20 | 94/155 | 1.231 | 0.161 |
| Total Observations | 0 | 0/20 | 0/159 |  | 0 | 0/18 | 0/127 | / | / |  | 0 | 0/20 | 0/155 | / | / |

All group compared with the negative control group, data are presented as mean ± SD. Statistical significance was indicated as follows: **P*＜0.01.

## Supplementary table 9. Incidence of skeletal malformations and variations in rat fetuses

| Parameters | Negative control group | | |  | Adjuvant control group | | |  | High dose group | | |
| --- | --- | --- | --- | --- | --- | --- | --- | --- | --- | --- | --- |
|  | Incidence(%) | Affected Litters | Affected Fetuses |  | Incidence(%) | Affected Litters | Affected Fetuses |  | Incidence(%) | Affected Litters | Affected Fetuses |
| Transposition of Umbilical Arteries | 0 | 0/20 | 0/152 |  | 5.6±23.6 | 1/18 | 1/122 |  | 0.6±2.5 | 1/20 | 1/144 |
| Thymic Horn | 6.0±10.0 | 6/20 | 9/152 |  | 6.7±9.3 | 7/18 | 9/122 |  | 4.9±8.4 | 6/20 | 7/144 |
| Dilated Right Renal Pelvis | 2.8±5.7 | 4/20 | 4/152 |  | 10.7±25.6 | 4/18 | 6/122 |  | 4.4±8.6 | 5/20 | 7/144 |
| Dilated Left Renal Pelvis | 1.3±4.1 | 2/20 | 2/152 |  | 9.5±23.5 | 6/18 | 6/122 |  | 3.1±6.6 | 4/20 | 5/144 |
| Kinked Ureter | 0.6±2.8 | 1/20 | 1/152 |  | 0 | 0/18 | 0/122 |  | 1.3±4.1 | 2/20 | 2/144 |
| Visceral Variations | 10.7±14.1 | 9/20 | 16/152 |  | 19.7±25.2 | 10/18 | 18/122 |  | 11.3±9.8 | 13/20 | 17/144 |
| Total Visceral Findings | 0 | 0/20 | 0/152 |  | 0 | 0/18 | 0/122 |  | 0 | 0/20 | 0/144 |
